# Supplementary material for: Phi 6 Bacteriophage Inactivation by Metal Salts, Metal Powders, and Metal Surfaces
Source: Viruses. 2022 Jan 21;14(2):204. doi: 10.3390/v14020204 (PMC8877498; doi:10.3390/v14020204)
Supplement: Supplementary file 1 [file viruses-14-00204-s001.zip › viruses-1554707-supplementary.pdf]

# Supplementarry material

**Phi 6 bacteriophage inactivation by metal salts, metal powders and metal surfaces**

Katja Molan, Ramin Rahmani, Daniel Krklec, Miha Brojan, David Stopar

## 1. Virus inactivation by physicochemical factors

The inactivation of Phi6 by temperature, pH, relative humidity (RH), sonication, and UV radiation are shown in Fig. S1. Phi6 phage was inactivated dramatically from 37 to 42 °C, with an 11 order of magnitude decrease in phage titer. The phage was stable in a pH range from 4 to 10. No infective phages were observed at pH less than 2 or greater than 12. Incubation of phage suspension at 33 °C at either 80 % or 10 % RH resulted in more than 4 logs drop in a viral titer, whereas at 28 °C the resultant drop was approximately 2 logs. The sonication efficiently reduced the viral titer. After 100 s of treatment, the viral titer decreased by more than 4 logs. With increased UV irradiation intensity viral titer decreased and at 400 mJ/cm$^2$ more than 4 log reduction was measured.

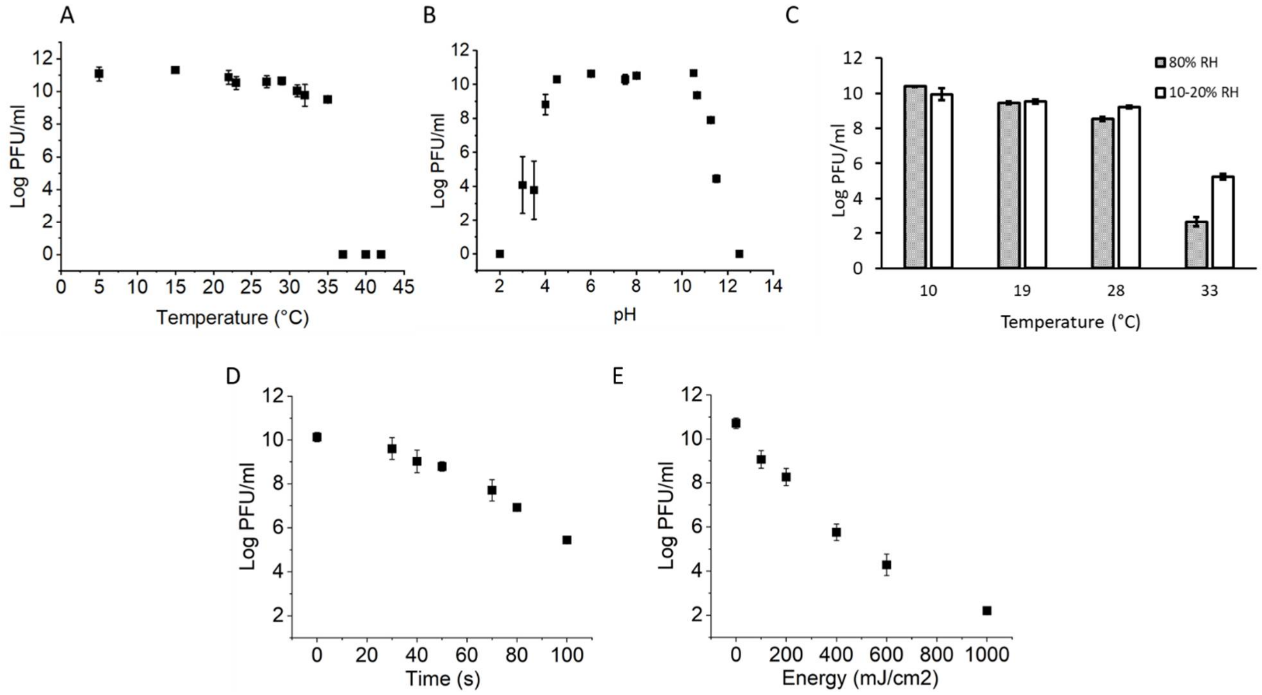

**Figure S1:** Effects of physicochemical factors on Phi6 inactivation. (A) temperature, (B) pH, (C) a combination of temperature and relative humidity (RH), (D) sonotrode sonication. (E) UV light. The average values and standard deviations are given (n ≥ 3).

## 2. Protocol optimization in sonication bath

Viral phage titer before and after sonication in a water bath was tested to determine the maximum sonication intensity and duration that did not affect viral titer (Table S1). A 5-minute sonication in degassing mode in water bath at 22 °C did not significantly changed viral titer and was used to test strongly attached viruses to different surfaces.

**Table S1:** Phi6 viral titer before and after treatment in Ultrasonic Bath. Viral titer (Log PFU/ml) for independent experiments is given with average number and standard deviation (n=4).

| **Untreated Phi6 – Control** (Log PFU/ml) | **Average Control** (Log PFU/ml ± SD) | **Treated Phi6 - Ultrasonic Bath, Degas mode, 5 min** (Log PFU/ml) | **Average Treated Phi6** (Log PFU/ml ± SD) |
|---|---|---|---|
| 10.7 | 10.6 ± 0.1 | 10.7 | 10.7 ± 0.2 |
| 10.7 | | 10.7 | |
| 10.5 | | 10.4 | |
| 10.5 | | 10.8 | |

## 3. Statistical analysis – ANOVA

Statistical analysis was performed for discs testing. Discs surfaces were numbered as:

***0 – control*** (Phi6 diluted in PBS, no contact with any surface)
***1 – steel***
***2 – $TiO_2$ anatase***
***11 – $TiO_2$+10%Ag 10%Cu***
***13 – Co28CrMo***
***14 – 45%$TiO_2$+5%Ag-45%$ZrO_2$+5%Cu***

**Table S2:** ANOVA for "Viruses in suspension".

**ANOVA – droplet analysis (viruses in suspension)**
Log PFU_ml

| | Sum of Squares | df | Mean Square | F | Sig. |
|---|---|---|---|---|---|
| Between Groups | 1086,104 | 5 | 217,221 | 2111,380 | <,001 |
| Within Groups | 7,922 | 77 | ,103 | | |

| Total | 1094,026 | 82 | | | |
|---|---|---|---|---|---|

# Post Hoc Tests

**Multiple Comparisons**

Dependent Variable: PFU_ml

Tukey HSD

| (I) Number | (J) Oznaka_Number | Mean Difference (I-J) | Std. Error | Sig. | 95% Confidence Interval Lower Bound | Upper Bound |
|---|---|---|---|---|---|---|
| ,00 - ***control*** | 1,00 | ,00800 | ,10564 | 1,000 | -,3007 | ,3167 |
| | 2,00 | ,36707* | ,12346 | **,044** | ,0062 | ,7279 |
| | 11,00 | 1,19920* | ,09463 | **<,001** | ,9226 | 1,4758 |
| | 13,00 | 10,25583* | ,14477 | **<,001** | 9,8327 | 10,6789 |
| | 14,00 | 10,25583* | ,13604 | **<,001** | 9,8582 | 10,6534 |
| 1,00 - ***steel*** | ,00 | -,00800 | ,10564 | 1,000 | -,3167 | ,3007 |
| | 2,00 | ,35907 | ,13704 | ,105 | -,0415 | ,7596 |
| | 11,00 | 1,19119* | ,11177 | **<,001** | ,8645 | 1,5179 |
| | 13,00 | 10,24782* | ,15651 | **<,001** | 9,7904 | 10,7053 |
| | 14,00 | 10,24782* | ,14848 | **<,001** | 9,8139 | 10,6818 |
| 2,00 - ***TiO2 anatase*** | ,00 | -,36707* | ,12346 | ,044 | -,7279 | -,0062 |
| | 1,00 | -,35907 | ,13704 | ,105 | -,7596 | ,0415 |
| | 11,00 | ,83212* | ,12874 | **<,001** | ,4558 | 1,2084 |
| | 13,00 | 9,88875* | ,16905 | **<,001** | 9,3947 | 10,3828 |
| | 14,00 | 9,88875* | ,16164 | **<,001** | 9,4163 | 10,3612 |
| 11,00 - ***TiO2+10%Ag 10%Cu*** | ,00 | -1,19920* | ,09463 | <,001 | -1,4758 | -,9226 |
| | 1,00 | -1,19119* | ,11177 | <,001 | -1,5179 | -,8645 |
| | 2,00 | -,83212* | ,12874 | <,001 | -1,2084 | -,4558 |
| | 13,00 | 9,05663* | ,14930 | **<,001** | 8,6203 | 9,4930 |
| | 14,00 | 9,05663* | ,14086 | **<,001** | 8,6449 | 9,4683 |
| 13,00 - ***Co28CrMo*** | ,00 | -10,25583* | ,14477 | <,001 | -10,6789 | -9,8327 |
| | 1,00 | -10,24782* | ,15651 | <,001 | -10,7053 | -9,7904 |
| | 2,00 | -9,88875* | ,16905 | <,001 | -10,3828 | -9,3947 |
| | 11,00 | -9,05663* | ,14930 | **<,001** | -9,4930 | -8,6203 |
| | 14,00 | ,00000 | ,17845 | 1,000 | -,5216 | ,5216 |
| 14,00 - ***45%TiO2+5%Ag-45%ZrO2+5%Cu*** | ,00 | -10,25583* | ,13604 | <,001 | -10,6534 | -9,8582 |
| | 1,00 | -10,24782* | ,14848 | <,001 | -10,6818 | -9,8139 |
| | 2,00 | -9,88875* | ,16164 | <,001 | -10,3612 | -9,4163 |
| | 11,00 | -9,05663* | ,14086 | <,001 | -9,4683 | -8,6449 |
| | 13,00 | ,00000 | ,17845 | 1,000 | -,5216 | ,5216 |

*. The mean difference is significant at the 0.05 level.

# Homogeneous Subsets

**PFU_ml**

Tukey HSD[a,b]

| Number | N | Subset for alpha = 0.05 1 | 2 | 3 |
|---|---|---|---|---|
| 13,00 | 6 | ,0000 | | |
| 14,00 | 7 | ,0000 | | |
| 11,00 | 20 | | 9,0566 | |
| 2,00 | 9 | | | 9,8888 |
| 1,00 | 14 | | | 10,2478 |
| ,00 | 27 | | | 10,2558 |
| Sig. | | 1,000 | 1,000 | ,108 |

Means for groups in homogeneous subsets are displayed.

a. Uses Harmonic Mean Sample Size = 10,361.

b. The group sizes are unequal. The harmonic mean of the group sizes is used. Type I error levels are not guaranteed.

**Table S3:** ANOVA for "Weakly and strongly attached viruses".

**ANOVA**

| | | Sum of Squares | df | Mean Square | F | Sig. |
|---|---|---|---|---|---|---|
| Swab | Between Groups | 94,522 | 4 | 23,630 | 76,035 | <,001 |
| | Within Groups | 11,810 | 38 | ,311 | | |
| | Total | 106,331 | 42 | | | |
| Sonication | Between Groups | 88,598 | 4 | 22,149 | 46,481 | <,001 |
| | Within Groups | 10,960 | 23 | ,477 | | |
| | Total | 99,558 | 27 | | | |

# Post Hoc Tests

**Multiple Comparisons**

Tukey HSD

| Dependent Variable | (I) Oznaka_Number | (J) Oznaka_Number | Mean Difference (I-J) | Std. Error | Sig. | 95% Confidence Interval Lower Bound | Upper Bound |
|---|---|---|---|---|---|---|---|
| **Swab** | 1,00 | 2,00 | ,55373 | ,24582 | ,183 | -,1501 | 1,2575 |
| | | 11,00 | 3,75855* | ,24582 | **<,001** | 3,0547 | 4,4624 |
| | | 13,00 | 2,52659* | ,27874 | **<,001** | 1,7285 | 3,3246 |
| | | 14,00 | 2,60990* | ,26513 | **<,001** | 1,8508 | 3,3690 |
| | 2,00 | 1,00 | -,55373 | ,24582 | ,183 | -1,2575 | ,1501 |
| | | 11,00 | 3,20481* | ,26280 | **<,001** | 2,4524 | 3,9572 |
| | | 13,00 | 1,97285* | ,29382 | **<,001** | 1,1316 | 2,8141 |
| | | 14,00 | 2,05617* | ,28094 | **<,001** | 1,2518 | 2,8605 |
| | 11,00 | 1,00 | -3,75855* | ,24582 | <,001 | -4,4624 | -3,0547 |
| | | 2,00 | -3,20481* | ,26280 | <,001 | -3,9572 | -2,4524 |
| | | 13,00 | -1,23196* | ,29382 | **,001** | -2,0732 | -,3907 |
| | | 14,00 | -1,14864* | ,28094 | **,002** | -1,9530 | -,3443 |
| | 13,00 | 1,00 | -2,52659* | ,27874 | <,001 | -3,3246 | -1,7285 |
| | | 2,00 | -1,97285* | ,29382 | <,001 | -2,8141 | -1,1316 |
| | | 11,00 | 1,23196* | ,29382 | ,001 | ,3907 | 2,0732 |
| | | 14,00 | ,08331 | ,31015 | ,999 | -,8047 | ,9713 |
| | 14,00 | 1,00 | -2,60990* | ,26513 | <,001 | -3,3690 | -1,8508 |
| | | 2,00 | -2,05617* | ,28094 | <,001 | -2,8605 | -1,2518 |
| | | 11,00 | 1,14864* | ,28094 | ,002 | ,3443 | 1,9530 |
| | | 13,00 | -,08331 | ,31015 | ,999 | -,9713 | ,8047 |
| **Sonication** | 1,00 | 2,00 | -,36805 | ,37281 | ,858 | -1,4701 | ,7340 |
| | | 11,00 | 3,27899* | ,42273 | **<,001** | 2,0294 | 4,5286 |
| | | 13,00 | -1,30721* | ,37281 | **,015** | -2,4092 | -,2052 |
| | | 14,00 | 3,31892* | ,42273 | **<,001** | 2,0693 | 4,5685 |
| | 2,00 | 1,00 | ,36805 | ,37281 | ,858 | -,7340 | 1,4701 |
| | | 11,00 | 3,64704* | ,44559 | **<,001** | 2,3299 | 4,9642 |
| | | 13,00 | -,93916 | ,39855 | ,164 | -2,1173 | ,2390 |
| | | 14,00 | 3,68697* | ,44559 | **<,001** | 2,3698 | 5,0042 |
| | 11,00 | 1,00 | -3,27899* | ,42273 | <,001 | -4,5286 | -2,0294 |
| | | 2,00 | -3,64704* | ,44559 | <,001 | -4,9642 | -2,3299 |
| | | 13,00 | -4,58620* | ,44559 | **<,001** | -5,9034 | -3,2690 |
| | | 14,00 | ,03993 | ,48812 | 1,000 | -1,4030 | 1,4828 |
| | 13,00 | 1,00 | 1,30721* | ,37281 | ,015 | ,2052 | 2,4092 |

| | | | | | | |
|---|---|---|---|---|---|---|
| | 2,00 | ,93916 | ,39855 | ,164 | -,2390 | 2,1173 |
| | 11,00 | 4,58620* | ,44559 | <,001 | 3,2690 | 5,9034 |
| | 14,00 | 4,62613* | ,44559 | <,001 | 3,3089 | 5,9433 |
| 14,00 | 1,00 | -3,31892* | ,42273 | <,001 | -4,5685 | -2,0693 |
| | 2,00 | -3,68697* | ,44559 | <,001 | -5,0042 | -2,3698 |
| | 11,00 | -,03993 | ,48812 | 1,000 | -1,4828 | 1,4030 |
| | 13,00 | -4,62613* | ,44559 | <,001 | -5,9433 | -3,3089 |

*. The mean difference is significant at the 0.05 level.

**Swab**

Tukey HSD[a,b]

| | | Subset for alpha = 0.05 | | |
|---|---|---|---|---|
| Number | N | 1 | 2 | 3 |
| 11,00 | 9 | 3,2450 | | |
| 14,00 | 7 | | 4,3937 | |
| 13,00 | 6 | | 4,4770 | |
| 2,00 | 9 | | | 6,4499 |
| 1,00 | 12 | | | 7,0036 |
| Sig. | | 1,000 | ,998 | ,284 |

Means for groups in homogeneous subsets are displayed.
a. Uses Harmonic Mean Sample Size = 8,129.
b. The group sizes are unequal. The harmonic mean of the group sizes is used. Type I error levels are not guaranteed.

**Sonication**

Tukey HSD[a,b]

| | | Subset for alpha = 0.05 | | |
|---|---|---|---|---|
| Number | N | 1 | 2 | 3 |
| 14,00 | 4 | ,4643 | | |
| 11,00 | 4 | ,5043 | | |
| 1,00 | 8 | | 3,7832 | |
| 2,00 | 6 | | 4,1513 | 4,1513 |
| 13,00 | 6 | | | 5,0905 |
| Sig. | | 1,000 | ,908 | ,216 |

Means for groups in homogeneous subsets are displayed.
a. Uses Harmonic Mean Sample Size = 5,217.
b. The group sizes are unequal. The harmonic mean of the group sizes is used. Type I error levels are not guaranteed.
